# *Porphyromonas gingivalis* Infection Induces Lipopolysaccharide and Peptidoglycan Penetration Through Gingival Epithelium

**DOI:** 10.3389/froh.2022.845002

**Published:** 2022-02-08

**Authors:** Hiroki Takeuchi, Eriko Nakamura, Shunsuke Yamaga, Atsuo Amano

**Affiliations:** ^1^Department of Preventive Dentistry, Osaka University Dental Hospital, Suita, Japan; ^2^Department of Preventive Dentistry, Graduate School of Dentistry, Osaka University, Suita, Japan

**Keywords:** periodontitis, *Porphyromonas gingivalis*, gingipains, gingival epithelium, lipopolysaccharide, peptidoglycan, epithelial barrier, tissue model

## Abstract

Periodontal diseases initiate on epithelial surfaces of the subgingival compartment, while the gingival epithelium functions as an epithelial barrier against microbial infection and orchestrates immune responses. *Porphyromonas gingivalis* is a major pathogen of periodontal diseases and has an ability to penetrate the epithelial barrier. To assess the molecular basis of gingival epithelial barrier dysfunction associated with *P. gingivalis*, we newly developed a three-dimensional multilayered tissue model of gingival epithelium with gene manipulation. Using this novel approach, *P. gingivalis* gingipains including Arg- or Lys-specific cysteine proteases were found to specifically degrade junctional adhesion molecule 1 and coxsackievirus and adenovirus receptor in the tissue model, leading to increased permeability for lipopolysaccharide, peptidoglycan, and gingipains. This review summarizes the strategy used by *P. gingivalis* to disable the epithelial barrier by disrupting specific junctional adhesion molecules.

## Significance Of *Porphyromonas gingivalis* Infection in Human Gingival Epithelium

Epithelial cells are located on the front line of infection defense, and function as a physical barrier against pathogenic bacteria and their products. The epithelial barrier is formed by cell-cell adhesion, and consists of tight junctions that prevent leakage of transported substances, and seal the paracellular pathway. Human gingival epithelial cells have been reported to express tight-junction associated proteins, such as claudin, occludin, junctional adhesion molecule 1 (JAM1), and zonula occludens-1 [1], among which JAM1, an immunoglobulin superfamily protein, reportedly localizes in mucosal epithelium of numerous organs [2].

Periodontitis is basically an infectious disease that causes destruction of periodontal tissues by interactions between periodontal pathogens and host cells [3]. Since gingival epithelial cells are the first to face periodontal pathogens, gingival epithelial tissues are potentially involved in the pathogenesis and progress of periodontitis. *Porphyromonas gingivalis*, a Gram-negative anaerobe, is a periodontal pathogen that expresses a variety of virulence factors, such as lipopolysaccharide (LPS), peptidoglycan (PGN), and gingipains. Periodontal diseases are multispecies infections involving pathogenic communities in which *P. gingivalis* can increase the pathogenicity of the entire multispecies periodontal community [3]. Various studies have shown that *P. gingivalis* occurrence is significantly associated with initiation of periodontitis, with odds ratios of 11.788 [4], 12.3 [5], and 5.6 [6] reported. It is also known that an increase in amount of “red complex” species, consisting of *P. gingivalis, Treponema denticola*, and *Tannerella forsythia*, in subgingival biofilm is related to initiation and progression of periodontitis [7]. A cross-sectional study revealed that *P. gingivalis* is the most influential pathogen among red complex bacteria [8]. However, it is ethically difficult to analyze the effects of *P. gingivalis* infection on tight junction-associated proteins using human gingival epithelium specimens. Hence, features of the physiological function related to tight junction-associated proteins in the oral cavity have become an interesting focus of research.

## Advantages Of 3D-Tissue Models Of Human Gingival Epithelium

From the standpoint of replacement, reduction, and refinement (3Rs), alternative methods for animal experiments are needed for medical research studies [9]. Physiological tissues are composed of various types of cells and connective tissues, thus how to construct three-tissue models with similar functions in living tissues has been investigated. Within an organism, the extracellular matrix has an important role to regulate the interface-surface structure of host cells. We previously reported that a cell-accumulation technique [10] using fibronectin and gelatin, extracellular matrixes, was useful to re-construct human gingival epithelial tissues [11, 12]. The advantages of this technique include (1) development of healthy human tissues, (2) gene manipulation including overexpression and knockdown, (3) direct measurement of fluorescent-tracer transmission in human tissues, (4) time-course observations of pathological condition before disease onset, and (5) administration of LPS and PGN, for examining PAMPs and infection by *P. gingivalis*. We have found that 3D-tissue models of gingival epithelium are useful for defining the cause-and-effect relationships of risk factors in terms of elimination of potential confounding factors.

## *P. gingivalis* Gingipains Degrade JAM1 AND CXADR

*P. gingivalis* secretes Arg- and Lys-specific cysteine proteases, termed Arg-gingipains (RgpA and RgpB) and Lys-gingipain (Kgp), respectively, which are major virulence factors [13, 14]. In a previous study, to clarify which tight junction-associated protein(s) are degraded by *P. gingivalis* infection, we infected immortalized human gingival epithelial (IHGE) cells [15] with *P. gingivlis* ATCC 33277 or KDP136, a Δ*kgp* Δ*rgpA* Δ*rgpB* mutant [16], for 1 h. Immunoblot and confocal microscopic analyses revealed that *P. gingivalis* apparently degraded JAM1 and coxsackievirus and adenovirus receptor (CXADR), another JAM-family protein [17], but not claudin 1, claudin 4, E-cadherin, occludin, or zonula occludens-1, in a gingipains-dependent manner [11, 12]. Notably, medium used for culturing *P. gingivalis* WT, but not that used for a Δ*kgp* Δ*rgpA* Δ*rgpB* mutant, also degraded JAM1 and CXADR of IHGE cells, indicating that gingipains function to degrade JAM1 and CXADR.

## Specific Degradation Of JAM1 And CXADR BY Gingipains

**Figure 1:**
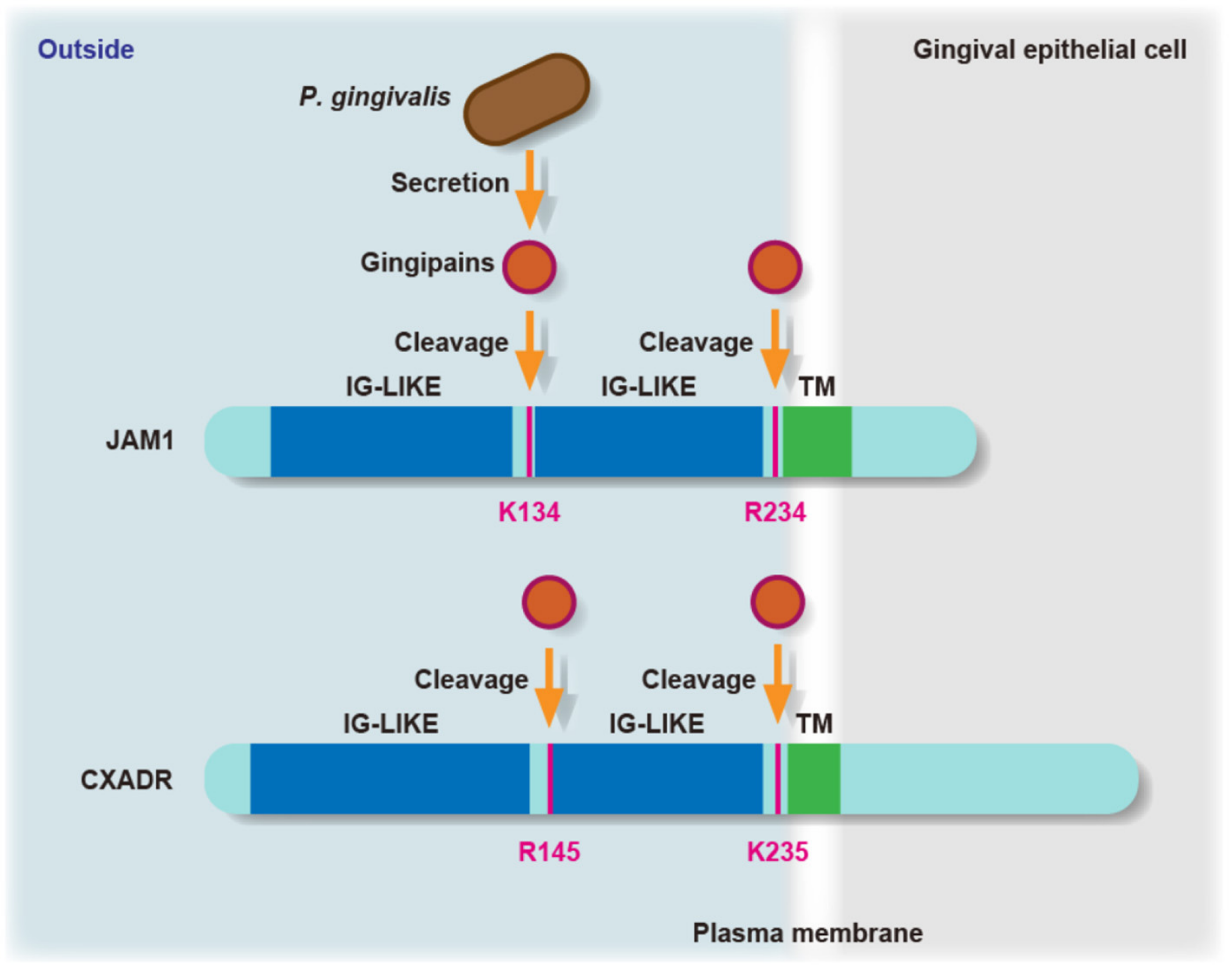
The residues involved in degradation of JAM1 and CXADR by *P. gingivalis* gingipains. Schematic view of the JAM1 and CXADR structure in gingival epithelial cells. The K134 and R234 residues of JAM1, and the R145 and K235 residues of CXADR are involved in degradation by *P. gingivalis* gingipains in gingival epithelial cells. IG-LIKE, immunoglobulin-like domain (blue); TM, transmembrane domain (green).

JAM family proteins have an extracellular domain along with two immunoglobulin-like domains, a single transmembrane domain, and a short cytoplasmic tail with a PDZ-domain-binding motif [18]. Hence, we constructed chimeric proteins of JAM1 and CXADR expressed by IHGE cells, and infected cells with *P. gingivalis*, after which the responsible residues of JAM1 K134 and R234, and CXADR R145 and K235 were examined for determining gingipains degradation (Figure 1). JAM1 K134 and CXADR R145 are located between the two immunoglobulin domains, while JAM1 R234 and CXADR K235 are set at the N-terminus of the transmembrane domain. A dimerization motif in the N-terminal immunoglobulin domain is essential for JAM1- or CXADR-homodimer formation, thus gingipains efficiently dampen the functions of JAM1 and CXADR.

*Fusobacterium nucleatum* and *Streptococcus gordonii* are human oral bacteria that can assemble mixed-species communities [19]. Hence, IHGE cells were infected with *F. nucleatum* or *S. gordonii*, and it was confirmed that the protein levels of JAM1 and CXADR were not decreased, thus indicating that *F. nucleatum* and *S. gordonii* do not degrade JAM1 and CXADR [11, 12].

Next, 3D-tissue models of gingival epithelium were constructed, and localization of JAM1 and CXADR was confirmed and found to be comparable to that seen in human gingiva. We also found that *P. gingivalis* WT decreased JAM1 and CXADR even in tissues 3–4 layers below the surface, whereas the Δ*kgp* Δ*rgpA* Δ*rgpB* mutant did not [11, 12]. These results indicate that gingipains continuously degrade JAM1 and CXADR, and deeply invade human gingival epithelial tissues.

## *P. gingivalis* Induces Penetration Of LPS and PGN Through Gingival Epithelium

LPS, a gram-negative bacteria endotoxin, and PGN, which exists in a mesh-like pattern outside the plasma membrane of most bacteria, are known as pathogen-associated molecular patterns (PAMPs) that cause initiation of host immune response [20]. In cases with leukocyte adhesion deficiency, one of the syndromes associated with periodontitis [21], LPS in the subepithelial area was reported to be detected in gingival tissues, but not in those from healthy cases [22]. In addition, plasma LPS levels were found to be correlated with multiple clinical parameters of aggressive periodontitis [23] and decreased by periodontal therapy [24]. Hence, we hypothesized that PAMPs from oral bacteria penetrate gingival epithelial tissues.

**Figure 2:**
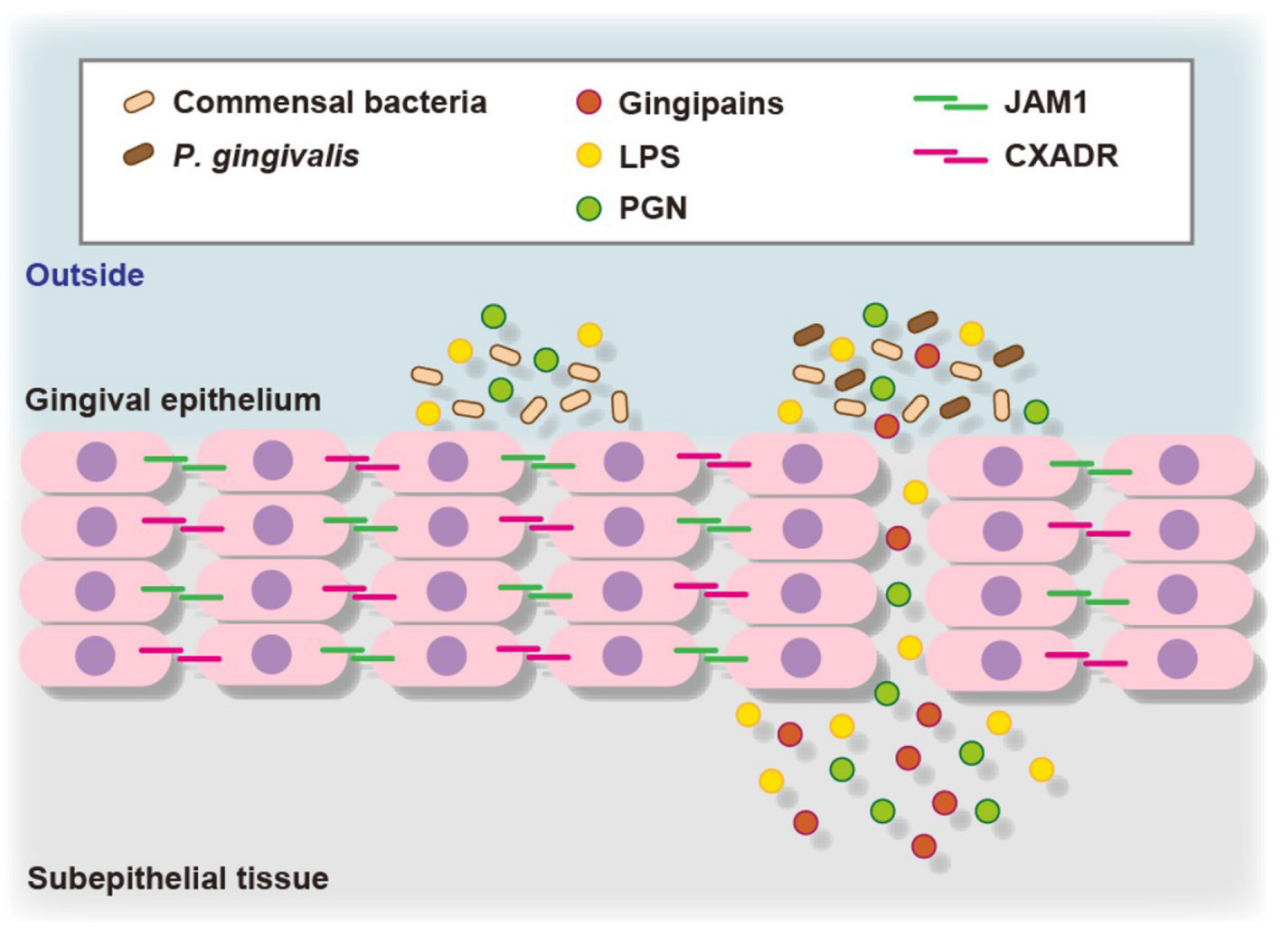
Proposed model of transfer of bacterial virulence factors by *P. gingivalis* gingipains through gingival epithelium. *P. gingivalis* gingipains degrade JAM1 and CXADR, leading to increased permeability to gingipains, LPS, and PGN. Subsequently, gingipains become translocated to deeper epithelium for additional degradation of JAM1 and CXADR, thus allowing LPS and PGN to penetrate the gingival epithelium and reach subepithelial tissues.

To assess the contribution of JAM1 expression to the permeability of gingival epithelial cells, 3D-tissue models of gingival epithelium were generated, then permeability assays were performed using fluorescent probe-tagged LPS or PGN in combination with *P. gingival* infection. To confirm the involvement of JAM1 or CXADR in *P. gingivalis*-affected permeability, 3D tissues were infected with *P. gingivalis* using IHGE cells overexpressing JAM1 or CXADR, which were then treated with fluorescent tracers [11, 12]. Thirty minutes after administration, the permeability to LPS or PGN was increased by *P. gingivalis* infection, whereas that was decreased by JAM1 or CXADR overexpression in gingival epithelial tissues. These results suggest that JAM1 and CXADR degradation by *P. gingivalis* causes penetration of gingival epithelium by LPS and PGN (Figure 2).

## Discussion

### Difference Between Gene Expression and Protein Localization

We confirmed that the immature forms of JAM1 and CXADR possessed a signal peptide and were localized in the endoplasmic reticulum [11, 12]. Generally, the levels of the immature forms of JAM1 and CXADR are proportional to those of the messenger RNA levels. In contrast, *P. gingivalis* degraded mature forms of JAM1 and CXADR in the plasma membrane, but not the immature forms in IHGE cells. These results suggest that surface protein localization of JAM1 and CXADR is needed to be confirmed in gingival epithelial cells to accurately evaluate the effects of risk factors of periodontitis.

### Protein Modification

It has been reported that JAM1 is phosphorylated at Y280 [25] and S284 [26], and glycosylated at N185 [27], while CXADR is glycosylated at N106 and N201 [28]. In general, protein phosphorylation modulates subcellular localization, and N-linked protein glycosylation is involved in cell–cell and cell–extracellular matrix attachment. Hence, elucidation of risk factors of periodontitis that have effects on JAM1- or CXADR-protein modification is considered to helpful to better understand its etiology.

### Intracellular Trafficking

The C-terminal cytosolic domain of JAM1 possesses a class II PDZ domain binding motif (-SFLV-COOH) [29]. In cytosolic space, JAM1 is known to associate with various partner proteins *via* the PDZ domain [30]. We confirmed that the immature forms of JAM1 and CXADR were localized in the endoplasmic reticulum, in which these proteins were apparently digested as a single peptide and N-glycosylated for maturation. To show biological activity, JAM family proteins must be transferred from the endoplasmic reticulum to plasma membrane, in which case regulator proteins may also bind with JAM *via* the PDZ domain. We recently observed that JAM1 localization in the plasma membrane was not disturbed by actin polymerization inhibitors (unpublished data). In contrast, actin depolymerization has been shown to disturb plasma-membrane localization of claudin-1 and occludin, tight junction proteins [31]. Thus, it is considered that transport of JAM family proteins occurs in a manner different from that of claudin and occludin.

### Other Cell Types

JAM was initially identified as a platelet membrane protein [32] and shown to play an important role in platelet assembly [33]. Generally, when blood-vessel walls are damaged, platelets aggregate in the wound and serve to stop bleeding. If the function of platelets is abnormal, a bleeding tendency will develop. To monitor the health or inflammation of gingival tissues, the parameter of bleeding on probing (BOP) has been well documented [34, 35]. Furthermore, a positive correlation between number of periodontal pockets with BOP and serum LPS concentration has been reported [36]. Thus, the molecular mechanisms related to how *P. gingivalis* affects platelets in the process of periodontal pathogenesis is quite interesting, though difficult to fully understand. Platelets are torn from the cytoplasm of polymorphonuclear giant cells in bone marrow and enter the bloodstream, and lose their nuclei. There are technical limitations when attempting to use human platelets for molecular biological research, such as passage culturing, gene manipulation, bacterial and viral contamination, and cross-contamination with the other cells, as well as confounding factors. Technical developments, including induction of differentiation to anuclear or short lifespan cells, as well as re-construction of human gingival epithelium, are needed in order to fully understand the etiology of periodontitis.

## Author Contributions

HT and AA wrote the manuscript. HT, EN, and SY constructed the images. All authors contributed to the article and approved the submitted version.

## Funding

This research was supported by JSPS KAKENHI Scientific Research grants (26253094 and 18H04068) (to AA), JSPS KAKENHI Grants-in-Aid for Young Scientists (15K20360 and 17K17083) (to HT), a JSPS KAKENHI Scientific Research grant (19K10085) (to HT), and a JSPS KAKENHI Grant-in-Aid for Young Scientists (Start-up) (21K21038) (to EN), each from the Japan Society for the Promotion of Science. The funders had no role in study design, data collection, preparation of the manuscript, or decision to publish.

## Conflict of Interest

The authors declare that the research was conducted in the absence of any commercial or financial relationships that could be construed as a potential conflict of interest.

## Publisher's Note

All claims expressed in this article are solely those of the authors and do not necessarily represent those of their affiliated organizations, or those of the publisher, the editors and the reviewers. Any product that may be evaluated in this article, or claim that may be made by its manufacturer, is not guaranteed or endorsed by the publisher.

## References

[1] Belibasakis GN Kast JI Thurnheer T Akdis CA Bostanci N. The expression of gingival epithelial junctions in response to subgingival biofilms. Virulence. (2015) 6:704–9. 10.1080/21505594.2015.1081731 26305580 PMC4720238

[2] Liang TW DeMarco RA Mrsny RJ Gurney A Gray A Hooley J. Characterization of huJAM1: evidence for involvement in cell-cell contact and tight junction regulation. Am J Physiol Cell Physiol. (2000) 279:C1733–43. 10.1152/ajpcell.2000.279.6.C1733 11078687

[3] Lamont RJ Koo H Hajishengallis G. The oral microbiota: dynamic communities and host interactions. Nat Rev Microl. (2018) 16:745–59. 10.1038/s41579-018-0089-x 30301974 PMC6278837

[4] Amano A Kuboniwa M Nakagawa S Akiyama S Morisaki I Hamada S. Prevalence of specific genotypes of *Porphyromonas gingivalis* fimA and periodontal health status. J Dent Res. (2000) 79:1664–8. 10.1177/00220345000790090501 11023261

[5] van Winkelhoff AJ Loos BG van der Reijden WA van der Velden U. *Porphyromonas gingivalis, Bacteroides forsythus* and other putative periodontal pathogens in subjects with and without periodontal destruction. J Clin Periodontol. (2002) 29:1023–8. 10.1034/j.1600-051X.2002.291107.x 12472995

[6] Torrungruang K Jitpakdeebordin S Charatkulangkun O Gleebbua Y. *Porphyromonas gingivalis, Aggregatibacter actinomycetemcomitans*, and *Treponema denticola* / *Prevotella intermedia* co-infection are associated with severe periodontitis in a Thai population. PLoS ONE. (2015) 10:e0136646. 10.1371/journal.pone.0136646 26313005 PMC4552424

[7] Socransky SS Haffajee AD Cugini MA Smith C Kent Jr RL. Microbial complexes in subgingival plaque. J Clin Periodontol. (1998) 25:124–44. 10.1111/j.1600-051X.1998.tb02419.x 9495612

[8] Chigasaki O Aoyama N Sasaki Y Takeuchi Y Mizutani K Ikeda Y. *Porphyromonas gingivalis*, the most influential pathogen in red-complex bacteria: a cross-sectional study on the relationship between bacterial count and clinical periodontal status in Japan. J Periodontol. (2021) 92:1719–29. 10.1002/JPER.21-0011 33856713

[9] Balls M. The origins and early days of the three Rs concept. Altern Lab Anim. (2009) 37:255–65. 10.1177/026119290903700306 19678726

[10] Nishiguchi A Yoshida H Matsusaki M Akashi M. Rapid construction of three-dimensional multilayered tissues with endothelial tube networks by the cell-accumulation technique. Adv Biomater. (2011) 23:3506–10. 10.1002/adma.201101787 21728193

[11] Takeuchi H Sasaki N Yamaga S Kuboniwa M Matsusaki M Amano A. *Porphyromonas gingivalis* induces penetration of lipopolysaccharide and peptidoglycan through the gingival epithelium *via* degradation of junctional adhesion molecule 1. PLoS Pathog. (2019) 15:e1008124. 10.1371/journal.ppat.1008124 31697789 PMC6932823

[12] Takeuchi H Yamaga S Sasaki N Kuboniwa M Matsusaki M Amano A. *Porphyromonas gingivalis* induces penetration of lipopolysaccharide and peptidoglycan through the gingival epithelium *via* degradation of coxsackievirus and adenovirus receptor. Cell Microbiol. (2021) 23:e13388. 10.1111/cmi.13388 34448537

[13] Nakayama K Kadowaki T Okamoto K Yamamoto K. Construction and characterization of arginine-specific cysteine protease (Arg-gingipain)-deficient mutants of *Porphyromonas gingivalis*. J Biol Chem. (1995) 270:23619–26. 10.1074/jbc.270.40.23619 7559528

[14] Potempa J Pike R Travis J. The multiple forms of trypsin-like activity present in various strains of *Porphyromonas gingivalis* are due to the presence of either Arg-gingipain or Lys-gingipain. Infect Immun. (1995) 63:1176–82. 10.1128/iai.63.4.1176-1182.1995 7890369 PMC173131

[15] Murakami S Yoshimura N Koide H Watanabe J Takedachi M Terakura M. Activation of adenosine receptor-enhanced iNOS mRNA expression by gingival epithelial cells. J Dent Res. (2004) 81:236–40. 10.1177/154405910208100403 12097306

[16] Shi Y Ratnayake DB Okamoto K Abe N Yamamoto K Nakayama K. Genetic analysis of proteolysis, hemoglobin binding, and hemagglutination of *Porphyromonas gingivalis*. Construction of mutants with a combination of rgpA, rgpB, kgp, and hagA. J Biol Chem. (1999) 274:17955–60. 10.1074/jbc.274.25.17955 10364243

[17] Ebnet K Suzuki A Ohno S Vestweber D. Junctional adhesion molecules (JAMs): more molecules with dual functions? J Cell Sci. (2004) 117:19–29. 10.1242/jcs.00930 14657270

[18] Weber C Fraemohs L Dejana E. The role of junctional adhesion molecules in vascular inflammation. Nat Rev Immunol. (2007) 7:467–77. 10.1038/nri2096 17525755

[19] Kolenbrander PE Anderson RN Blehert DS Egland PG Foster JS Palmer RJ Jr. Communication among oral bacteria. Microbiol Mol Biol Rev. (2002) 66:486–505. 10.1128/MMBR.66.3.486-505.2002 12209001 PMC120797

[20] Kawai T Akira S. Toll-like receptors and their crosstalk with other innate reeptors in infection and immunity. Immunity. (2011) 34:637–50. 10.1016/j.immuni.2011.05.006 21616434

[21] Albander JM Susin C Hughes FJ. Manifestations of systemic diseases and conditions that affect the periodontal attachment apparatus: Case definitions and diagnostic considerations. J Periodontol. (2018) 89(Suppl 1): S183–203. 10.1002/JPER.16-0480 29926941

[22] Moutsopoulos NM Chalmers NI Barb JJ Abusleme L Greenwell-Wild T Dutzan N. Subgingival microbial communities in leukocyte adhesion deficiency and their relationship with local immunopathology. PLoS Pathog. (2015) 11:e1004698. 10.1371/journal.ppat.1004698 25741691 PMC4351202

[23] Shaddox LM Wiedey J Calderon NL Magnusson I Bimstein E Bidwell JA. Local inflammatory markers and systemic endotoxin in aggressive periodontitis. J Dent Res. (2011) 90:1140–4. 10.1177/0022034511413928 21730256 PMC3169885

[24] Kalash D Vovk A Huang H Aukhil I Wallet SM Shaddox LM. Influence of periodontal therapy on systemic lipopolysaccharides in children with localized aggressive periodontitis. Pediatr Dent. (2015) 37:35–40. 26531074 PMC4634009

[25] Fan S Weight CM Lussint AC Hilgarth RS Brazil JC Ettel M. Role of JAM-A tyrosine phosphorylation in epithelial barrier dysfunction during intestinal inflammation. Mol Biol Cell. (2019) 30:566–78. 10.1091/mbc.E18-08-0531 30625033 PMC6589701

[26] Ozaki H Ishii K Arai H Horiuchi H Kawamoto T Suzuki H. Junctional adhesion molecule (JAM) is phosphorylated by protein kinase C upon platelet activation. Biochem Biophys Res Commun. (2000) 276:873–8. 10.1006/bbrc.2000.3574 11027562

[27] Scott DW Tolbert CE Graham DM Wittchen E Bear JE Burridge K. N-glycosylation controls the function of junctional adhesion molecule-A. Mol Biol Cell. (2015) 26:3205–14. 10.1091/mbc.e14-12-1604 26224316 PMC4569312

[28] Excoffon KJ Gansemer N Traver G Zebner J. Functional effects of coxsackievirus and adenovirus receptor glycosylation on homophilic adhesion and adenoviral infection. J Virol. (2007) 81:5573–8. 10.1128/JVI.02562-06 17376928 PMC1900266

[29] Songyang Z Fanning AS Fu C Xu J Marfatia SM Chishti AH. Recognition of unique carboxyl-terminal motifs by distinct PDZ domains. Science. (1997) 275:73–7. 10.1126/science.275.5296.73 8974395

[30] Steinbacher T Kummer D Ebnet K. Junctional adhesion molecule-A: functional diversity through molecular promiscuity. Cell Mol Life Sci. (2018) 75:1393–409. 10.1007/s00018-017-2729-0 29238845 PMC11105642

[31] Shen L Turner JR. Actin depolymerization disrupts tight junctions *via* caveolae-mediated endocytosis. Mol Biol Cell. (2005) 16:3919–36. 10.1091/mbc.e04-12-1089 15958494 PMC1196308

[32] Kornecki E Walkowiak B Naik UP Ehrlich YH. Activation of human platelets by a stimulatory monoclonal antibody. J Biol Chem. (1990) 265:10042–8. 10.1016/S0021-9258(19)38776-9 2351647

[33] Sobocka MB Sobocki T Banerjee P Weiss C Rushbrook JI Norin AJ. Cloning of the human platelet F11 receptor: a cell adhesion molecule member of the immunoglobulin superfamily involved in platelet aggregation. Blood. (2000) 95:2600–9. 10.1182/blood.V95.8.2600 10753840

[34] Lang NP Joss A Tonetti MS. Monitoring disease during supportive periodontal treatment by bleeding on probing. Periodontol 2000. (1996) 12:44–8. 10.1111/j.1600-0757.1996.tb00080.x 9567993

[35] Lang NP Bartold PM. Periodontal health. J Periodontol. (2018) 89(Suppl 1):S9–16. 10.1002/JPER.16-0517 29926938

[36] Pussinen PJ Vilkuna-Rautiainen T Alfthan G Palosuo T Jauhiainen M Sundvall J. Severe periodontitis enhances macrophage activation *via* increased serum lipopolysaccharide. Arterioscler Thromb Vasc Biol. (2004) 24:2174–80. 10.1161/01.ATV.0000145979.82184.9f 15388525

